# Glycosylated Biotherapeutics: Immunological Effects of N-Glycolylneuraminic Acid

**DOI:** 10.3389/fimmu.2020.00021

**Published:** 2020-01-23

**Authors:** Sharon Yehuda, Vered Padler-Karavani

**Affiliations:** Department of Cell Research and Immunology, The George S. Wise Faculty of Life Sciences, Tel Aviv University, Tel Aviv, Israel

**Keywords:** antibody, biotherapeutics, glycosylation, sialic acid, *N*-glycolylneuraminic acid (Neu5Gc), immunology, anti-carbohydrate antibodies

## Abstract

The emerging field of biotherapeutics provides successful treatments for various diseases, yet immunogenicity and limited efficacy remain major concerns for many products. Glycosylation is a key factor determining the pharmacological properties of biotherapeutics, including their stability, solubility, bioavailability, pharmacokinetics, and immunogenicity. Hence, an increased attention is directed at optimizing the glycosylation properties of biotherapeutics. Currently, most biotherapeutics are produced in non-human mammalian cells in light of their ability to produce human-like glycosylation. However, most mammals produce the sialic acid *N*-glycolylneuraminic acid (Neu5Gc), while humans cannot due to a specific genetic defect. Humans consume Neu5Gc in their diet from mammalian derived foods (red meat and dairy) and produce polyclonal antibodies against diverse Neu5Gc-glycans. Moreover, Neu5Gc can metabolically incorporate into human cells and become presented on surface or secreted glycans, glycoproteins, and glycolipids. Several studies in mice suggested that the combination of Neu5Gc-containing epitopes and anti-Neu5Gc antibodies could contribute to exacerbation of chronic inflammation-mediated diseases (e.g., cancer, cardiovascular diseases, and autoimmunity). This could potentially become complicated with exposure to Neu5Gc-containing biotherapeutics, bio-devices or xenografts. Indeed, Neu5Gc can be found on various approved and marketed biotherapeutics. Here, we provide a perspective review on the possible consequences of Neu5Gc glycosylation of therapeutic protein drugs due to the limited published evidence of Neu5Gc glycosylation on marketed biotherapeutics and studies on their putative effects on immunogenicity, drug efficacy, and safety.

## Introduction

Biotherapeutics are a rapidly increasing portion of the pharmaceutical market, with over a 100 new products approved and marketed in the U.S. and the European Union over the past decade (1). Among the commonly used biotherapeutics are antibodies, cytokines, enzymes, and hormones, originally purified from living organisms and characterized by their therapeutic potential, with limited evaluation of their potential immunological effects in recipient patients. Large-scale manufacturing of these therapeutic products involves expression of recombinant DNA in biological systems such as bacteria, yeast, insect and mammalian cells, as well as transgenic animals (1, 2). Being produced in living systems, therapeutic proteins often undergo post-translational modifications, most notably glycosylation.

Glycosylation is an important and ubiquitous modification, in which sugar chains (glycans) are covalently attached to proteins or lipids. Glycan biosynthesis is a template-independent process, which rely on a complex network of serially operating glycan-modifying enzymes (3, 4). The variety of possible monosaccharide compositions and modifications, linkage configurations and branching points gives rise to a tremendous diversity of glycan structures (Varki et al., 2015). Since this is not a template-driven process, proteins with identical amino acid sequences would typically differ in the degree of occupancy of their glycosylation sites (macro-heterogeneity), and would carry different glycans in a specific glycosylation site (micro-heterogeneity) (5). The glycosylation pattern of a cell changes through development and differentiation, under different environmental conditions, and during pathologies such as inflammation and malignancy, indicating the involvement of glycans in numerous processes in physiology and in disease (6).

**Figure 1:**
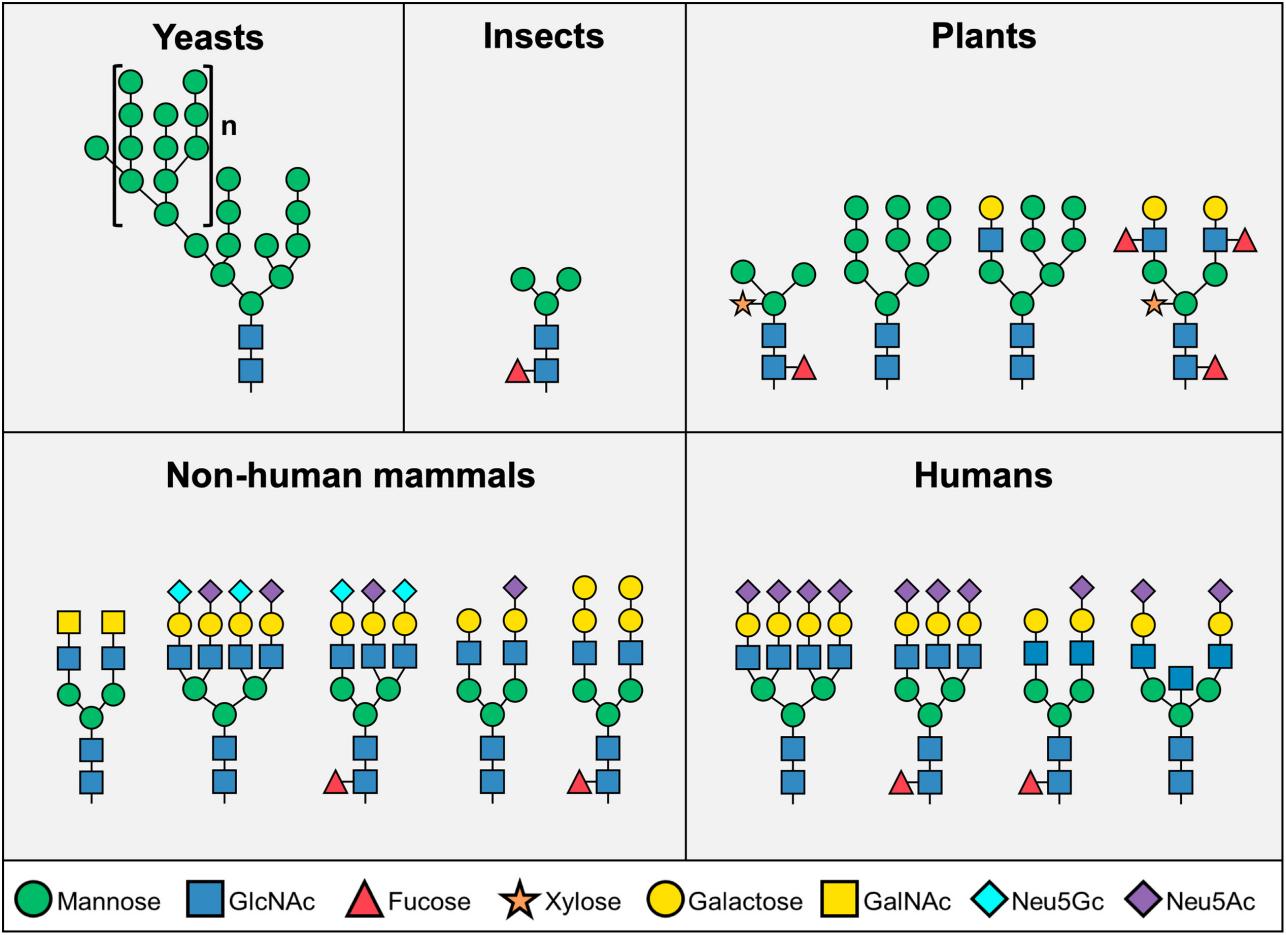
Representative *N*-glycosylation pattern produced in different organisms. *N*-linked glycosylation process starts with biosynthesis of a common core structure of Man3GlcNAc2, but additional modifications varies significantly among species. Yeast cells typically produce high-mannose type glycans, while most insect *N*-glycans are composed of the core structure, and to a lower extent, additional mannose, fucose, and galactose. Plant cells produce more complex type glycans, often containing xylose. Mammalian cells mainly synthesize “human-like” complex type *N*-glycans, however human cells do not express the two common mammalian glycan antigens αGal and Neu5Gc (Man – mannose; GlcNAc – *N*-acetylglucosamine; GalNAc – *N*-acetylgalactosamine; Neu5Gc – *N*-glycolylneuraminic acid; Neu5Ac – *N*-Acetylneuraminic acid).

Glycosylation of biotherapeutics has a substantial impact on their pharmacological properties and biological activity (7–10). Biotherapeutics glycosylation is largely determined by their production system (Figure 1). While non-mammalian cells (i.e., yeast, insect, or plant cells) are attractive due to their high yields, production of most biopharmaceutical products have shifted into mammalian expression platforms (i.e., hamster, human, or mouse cells) largely owing to consideration of their different glycosylation patterns (1). While yeast cells contain mostly high-mannose structures, mammalian-derived systems carry more complex glycans that include galactose, fucose and sialic acids (Figure 1)—all dramatically affecting the pharmacodynamics and pharmokinetics of the drugs, most notable in glycosylated-antibodies (11–13). Higher levels of sialic acid at the tips of glycan chains generally improves serum half-life and stability of biotherapeutics (12, 14, 15), partly since in the presence of terminal sialic acid glycosylated-biotherapeutics are not recognized by liver asialoglycoprotein receptors (ASGR1) or mannose receptors (MR; CD206), thereby preventing their rapid removal from circulation (12, 16). In addition, the negative charge of sialic acids positively contribute to their thermal stability and solubility (17, 18). Monoclonal antibodies constitute a major class of biotherapeutics, and in many of these antibodies the functionality is directly regulated by the glycosylation on their Fc domain. All IgG antibodies are glycosylated at a conserved asparagine residue (Asn297) in the Fc region (19), and some are also glycosylated at their Fab region (20–22). The glycan on Asn297 site modulates the shape of the Fc domain in a way that it alters its ability to interact with various Fc receptors (10, 15, 20, 23, 24). Remarkably, IgG Fc glycosylation is altered in pathological conditions such as autoimmunity (25), infection (10), and cancer (26–28), thereby modulating their effector functions (29). Interestingly, removal of the whole *N*-glycan revokes the ability of the Fc domain to interact with Fc receptors, thus Fc glycosylation is essential for the IgG effector functions (13, 30). The absence of fucose residues enhances antibody-dependent cellular cytotoxicity (31). In addition, higher presence of galactose promotes complement-dependent cytotoxicity, while decreased galactosylation leads to alternative complement cascade activation (32, 33). IgG antibodies with higher amount of terminal α2–6-linked sialic acids are recognized by DC-SIGN on dendritic cells, leading to anti-inflammatory activity (34, 35), while on the other hand activation of dendritic cells through antibody aggregates may induce immunogenicity and development of anti-drug antibodies (36). Aiming to optimize glycosylation properties, currently most biotherapeutics are produced in mammalian expression systems, with their ability to produce human-like glycosylation (1, 2, 37). Major efforts had been put into various methods for cell-glycoengineering to control antibody glycosylation (1, 35, 38–40), or to predict the glycosylation based on computational modeling (13, 38, 41–44).

Although humans and most other mammals have relatively similar glycosylation patterns, two major differences have been identified. Unlike most other mammals, humans lack the enzymes to synthesize the Galα1–3Galβ1–(3)4GlcNAc (αGal) epitope and the common sialic acid *N*-glycolylneuraminic acid (Neu5Gc) (45) (Figure 1). In addition to the inability to naturally express these sugar structures, all humans produce circulating antibodies against both antigens (45–49). In contrast to αGal, exogenous Neu5Gc can be metabolically incorporated into newly synthesized glycans and become presented on human cells (50, 51). Co-existence of Neu5Gc-containing epitopes and circulating anti-Neu5Gc antibodies have been suggested to exacerbate chronic inflammation-mediated diseases (52–57). This immune-conflict may be further complicated with exposure to Neu5Gc-containing biotherapeutics, bio-devices or xenografts. Indeed, recent studies have suggested that Neu5Gc-glycans have an enormous diversity (58–60), and predicted to be widely found on various approved and marketed biotherapeutics (2, 61), such as Cetuximab (61) and anti-thymocyte globulin (62–65). Although biotherapeutics provide effective treatment for a variety of clinical conditions, suboptimal efficacy and safety are major concerns for many of these products. Herein, we discuss the unique situation of Neu5Gc-containing biotherapeutics in the face of anti-Neu5Gc responses in humans, and the current knowledge on the effects of Neu5Gc on immunogenicity, efficacy, and safety of biotherapeutics.

## Neu5Gc is Immunogenic in Humans

Sialic acids are 9-carbon α-keto acidic sugars usually present at the outermost part of glycans in animals (5, 66). The two most common sialic acids in mammals are *N*-acetylneuraminic acid (Neu5Ac) and its hydroxylated form, Neu5Gc. Conversion of CMP-Neu5Ac to CMP-Neu5Gc is catalyzed by the enzyme CMP-*N*-acetylneuraminic acid hydroxylase (CMAH) that is inactive in humans (66). In contrast to all other mammals, humans cannot synthesize Neu5Gc due to irreversible mutation in the *CMAH* gene that occurred ~3 million years ago, before the appearance of the genus *Homo* (67–70). Nevertheless, consumption of Neu5Gc-containing mammalian-derived products (e.g., red meat and dairy) results in uptake of Neu5Gc-glycoproteins through micropinocytosis (71–73) and metabolic incorporation of Neu5Gc epitopes into newly synthesized glycans (50, 56, 72–74). Thus, low levels of Neu5Gc are present in human tissues, mostly on endothelium and epithelium, and are known to accumulate in certain pathological conditions, mostly in cancer (52, 56, 71, 75).

This unique phenomenon results in presentation of foreign antigen in the context of self (Neu5Gc is replacing the self Neu5Ac on existing cellular glycans), termed “Xeno-autoantigen” (47, 57). Hence, Neu5Gc is foreign in humans and mediates production of a complex anti-Neu5Gc antibodies response, or “Xeno-autoantibodies” (47, 51, 57, 76). Neu5Gc is a 325 Dalton molecule and cannot by itself fill the paratope of an antibody, yet Neu5Gc-containing glycan-epitopes are highly diverse (58–60) and are recognized by polyclonal anti-Neu5Gc IgM, IgA, and mostly IgG antibodies that make up 0.1–0.2% of total circulating antibodies in humans (47, 49, 77–79). Anti-Neu5Gc antibodies in humans arise already in infants, soon after the introduction of dietary Neu5Gc (e.g., cow milk in baby formula, meat-containing grinded foods), and have been suggested to be induced through uptake of dietary Neu5Gc by non-typeable *Haemophilus influenzae* (NTHi) during infection in infants (80), and through micropinocytosis of Neu5Gc-glycoproteins into human cells followed by recycling into the cells surface glycoproteins and glycolipids (71–74). In fact, all healthy humans examined thus far had anti-Neu5Gc antibodies, although sometimes at low levels and with limited repertoires (47, 49, 78, 81). This antibody response against Neu5Gc can be higher in certain pathologies and may remain high for decades (82–84).

Studies in mice had suggested that the co-existence of Neu5Gc-glycans and serum anti-Neu5Gc antibodies may lead to immune-driven chronic inflammation, termed “xenosialitis,” thereby exacerbating chronic inflammation-related diseases such as cancer, cardiovascular disease and autoimmunity (52, 53, 57, 84–86). For example, high anti-Neu5Gc IgG titers were shown to be associated with increased risk for colorectal cancer (84), which also fits the reported association of red meat consumption and higher carcinoma risk (55, 87–89). Similarly, in a human-like mouse model (*Cmah*-KO) high consumption of Neu5Gc resulted in an inflammatory phenotype, and together with circulating anti-Neu5Gc antibodies (in *Cmah*/*Ldlr*-DKO mouse model) resulted in increased atherosclerosis (52, 86, 90). These findings in mice fit the reported high risk of cardiovascular disease that is associated with consumption of red meat and processed meat (91, 92), although clear evidence in humans is still controversial, at least through *in vitro* studies on effects of anti-Neu5Gc antibodies on human endothelial cells that express authentic Neu5Gc levels (65). Neu5Gc and anti-Neu5Gc antibodies had also been suggested to participate in autoimmunity (54, 55, 93). Altogether, this unique human-specific immune conflict could help explain the susceptibility to numerous chronic inflammation-related diseases, which conspicuously occur in humans (94). The consequences of Neu5Gc/anti-Neu5Gc responses in humans could potentially be further exacerbated by exposure to Neu5Gc-containing biotherapeutics, bio-devices, or xenografts.

## Neu5Gc on Marketed Biotherapeutics Associated With Their Production System

Production of many biotherapeutics involves non-human mammalian cells, serum or serum-derived substances, thus are likely to contain some levels of Neu5Gc. Generalizations cannot be made since glycosylation properties, including Neu5Gc levels, are influenced by many factors during the production process. Yet, it is reasonable to assume the relative Neu5Gc levels in biotherapeutics according to their production systems (2). The most common platform for biotherapeutics is Chinese hamster ovary (CHO) cells (1, 2, 95). Several studies have suggested the presence of Neu5Gc on biotherapeutics produced in CHO cells, though in relatively low levels (2, 95, 96). Baby hamster kidney cells (BHK-21) are also often used for production of biotherapeutics and are expected to express low levels of Neu5Gc (2). By contrast, murine myeloma cell lines (e.g., NS0 and Sp2/0) are known to produce Neu5Gc at significantly higher levels (2, 97, 98). Drugs produced in animals (non-human mammals that are known to synthesize Neu5Gc intrinsically; e.g., cow, pig, goat, sheep, and rabbit) are also likely to contain Neu5Gc, since they were shown to express high amounts of Neu5Gc (50, 60). For example, antithrombin produced in goat milk and anti-thymocyte globulin derived from rabbit, are known to contain high levels of Neu5Gc (2, 62, 63). Similarly, Neu5Gc is also widely found in xenografts that are used for organ and tissue replacement in humans, as demonstrated with tissues derived from cows and pigs (99–102). These findings also prompted the generation of Neu5Gc-deficient animals as novel platforms (103–107).

Human cell lines represent the ideal production platform in terms of glycosylation properties, but their high risk of viral transmission and low protein yield make them less popular for production of biotherapeutics (37). Nevertheless, several products derived from human cells (HEK293 and HT-1080) have been approved in recent years (1). Utilization of these cells may become more common in the future, yet the presence of Neu5Gc in their products remains a significant concern, as it can also be metabolically incorporated from exogenous sources (i.e., from the growth media). Hence, even human cells can produce Neu5Gc-containing biotherapeutics if Neu5Gc is unintentionally supplemented in their growth media, for example through the addition of animal serum or serum-derived substances (2, 45). Although it was well-known that humans cannot express Neu5Gc, its immunogenic potential was under-rated for years, and accordingly its presence on biotherapeutics was largely disregarded. With the accumulating information about Neu5Gc and anti-Neu5Gc antibodies in humans, the presence of Neu5Gc on biotherapeutics should be re-evaluated. While the effect of Neu5Gc on biotherapeutics remains poorly characterized, several recent studies addressed possible consequences (61–65, 108–111), as described below.

## Effects on Serum Anti-Neu5Gc IgG Responses in Humans

Treatment of human patients with Neu5Gc-containing biotherapeutics can significantly alter the pre-existing immune response against Neu5Gc, both quantitatively and qualitatively. Yet, some studies failed to show human immune response against Neu5Gc-containing biotherapeutics, as in the case of recombinant erythropoietin that was produced in CHO cells (96, 112). Of note, these conclusions were based on the human response evaluated against Neu5Gc-containing gangliosides. It is possible that with current technologies as glycan microarrays it would be possible to revisit these findings. More recent studies were able to clearly demonstrate immunological effects in humans. Anti-thymocyte globulin (ATG) is an immunosuppressive biotherapeutic commonly used in transplantation and autoimmune diseases (113). ATG is a polyclonal IgG produced in rabbits and was shown to contain Neu5Gc (62, 63). One of the side effects during treatment with this drug is the development of an immune reaction against the non-human animal-derived immunoglobulins. This is characterized by immune complex formation that can develop into a serum sickness disease (62, 114). In fact, without strong immunosuppression most patients will develop serum sickness (114). Furthermore, it was shown that ~10% of first-kidney graft recipients treated with the immunosuppressive drug ATG developed serum sickness disease, and in addition had increased serum anti-Neu5Gc IgG responses (62). The serum sickness was associated with late graft loss, and these patients exhibited further elevated titers of anti-drug and anti-Neu5Gc IgG in blood samples >4 years post-transplantation compared to patients without serum sickness (62). In another study, ATG treatment was found to be associated with a shift in anti-Neu5Gc IgG repertoire in transplantation patients over time (64). Similarly, analyzing early events in another prospective study of kidney-graft recipients within their first year showed that patients with ATG induction treatment had a highly significant increase in anti-Neu5Gc IgG levels compared to patients not treated with ATG. In addition, these antibodies shifted their response repertoire over time to recognize different Neu5Gc-glycans, even in the face of a strong immunosuppression in those patients, but no effect on the graft function was observed within the limit of this study (110).

While mostly used in transplantation, ATG therapy was also explored as a therapeutic drug in young adults within the Study of Thymoglobulin to arrest Type 1 Diabetes (START clinical trial) (114). In these diabetic patients, ATG treatment also resulted in a highly significant increase in levels of serum anti-Neu5Gc IgM and IgG that peaked after 1 month and remained detectable even 1 year after treatment (108). Further characterization of the top responders by elaborated glycan microarrays demonstrated the rapid increase in responses against multiple Neu5Gc-glycans after 1 month, persistence over 2 years, and further demonstrated altered repertoires of serum anti-Neu5Gc IgG (63). In fact, ATG treatment changed the pre-existing response to induce anti-Neu5Gc IgG of higher affinity with extended diversity. Interestingly, in some patients there was *de-novo* recognition of various Neu5Gc-containing glycan epitopes, including of Neu5Gc-glycans normally expressed on glycolipids that were not present on the ATG drug (63). Overall, these findings suggested that Neu5Gc-containing biotherapeutics are immunogenic reagents, and once injected into humans that already express circulating anti-Neu5Gc antibodies, act as triggers of extended immune responses. In fact, current data support their role as inducers of secondary anti-Neu5Gc immune responses. In some individuals possibly also triggering a recall of memory responses inducing antibody recognition of Neu5Gc-glycans that had not been presented on the drug.

## Anti-Neu5Gc Antibodies in Disease

It was postulated that such elevated anti-Neu5Gc responses could potentially increase Neu5Gc-mediated xenosialitis and chronic inflammation-mediated diseases, as cancer and atherosclerosis (53). High pre-existing total anti-Neu5Gc IgG levels measured by glycan microarrays were associated with increased risk of colorectal cancer (in a cohort of 71 colorectal cancer cases and matched controls of the EPIC-Norfolk cohort plasma samples) (84). However, based on a large cohort of ~200,000 kidney allograft recipients, average anti-Neu5Gc IgG responses measured by EIA method did not show increased colon cancer risk in the ~18% ATG-treated patients compared to those not treated with ATG (111). Of note, these studies evaluated different pools of blood anti-Neu5Gc IgG antibodies and measured by different methods: pre-existing antibodies by arrays (84) vs. drug-induced antibodies by EIA (111). Currently, different methods are available to measure anti-Neu5Gc antibody responses (49, 115), and there are clear differences between pre-existing vs. ATG-induced anti-Neu5Gc IgG (63, 65), that together could perhaps explain the different analysis outcome regarding cancer risk.

Likewise, contradicting reports exist regarding anti-Neu5Gc antibodies in the context of cardiovascular disease risks. Aiming to examine gene expression profiles by *in vitro* studies, human endothelial cells that were engineered to express low levels of surface Neu5Gc (mimicking the levels expected to be present from dietary intake in humans) were exposed to different pools and dose of affinity-purified anti-Neu5Gc antibodies. This analysis showed differential gene expression when cells were exposed to ATG-induced compared to pre-existing anti-Neu5Gc antibodies or in the absence of such antibodies. Interestingly, drug-induced anti-Neu5Gc antibodies did not significantly upregulate inflammation-related genes that would be expected in xenosialitis (65). However, other *in vivo* studies in the human-like Neu5Gc-deficient mice also lacking the LDL receptor showed increased atherosclerosis propensity only when both high levels of diet-derived Neu5Gc-antigens and induced anti-Neu5Gc antibodies were present, thus supporting xenosialitis (90). Altogether, these findings suggest that anti-Neu5Gc antibody responses in humans are complex and further studies are needed to better understand their relationship with various diseases in humans.

## Rapid Clearance of Neu5Gc-Containing Biotherapeutics *in vivo*—Evidence in Mice

**Figure 2:**
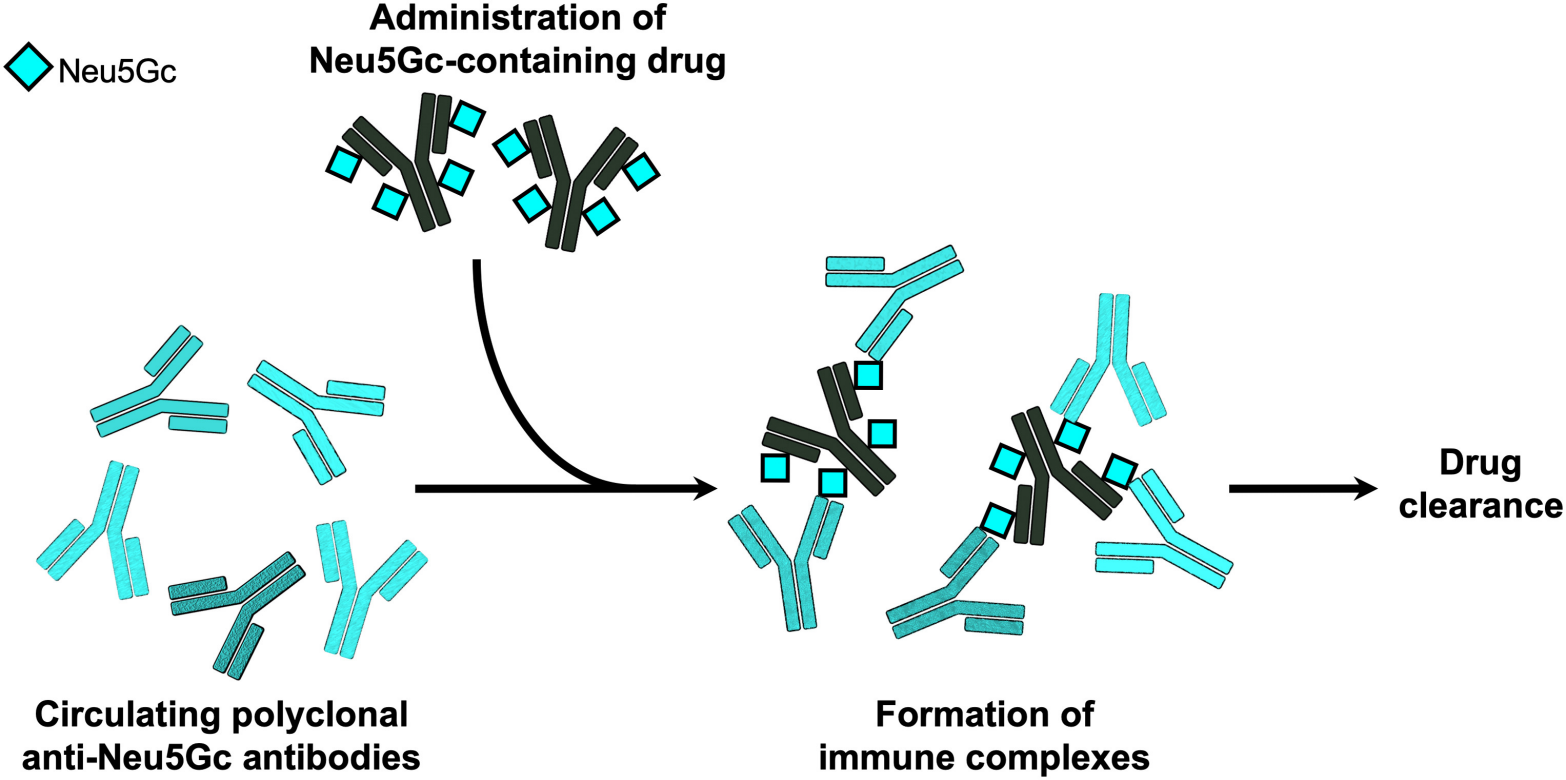
Immune complexes of Neu5Gc-containing biotherapeutics. In the human-like Neu5Gc-deficient *Cmah*-KO mice, it was demonstrated that circulating polyclonal anti-Neu5Gc antibodies can bind Neu5Gc-containing biotherapeutic monoclonal antibodies and generate immune complexes that mediated rapid clearance of the biotherapeutic drug (61).

Besides the immunogenicity of Neu5Gc on biotherapeutics, it was postulated that these Neu5Gc-drugs could potentially be recognized by circulating anti-Neu5Gc antibodies in humans, and by that affect drug levels and/or efficacy in patients. This was directly investigated using the top selling cancer biotherapeutic monoclonal antibodies targeting EGFR (61). Consistent with their production system, it was shown that Cetuximab produced in murine myeloma cells contains Neu5Gc, while Panitumumab expressed in CHO cells lack Neu5Gc (61). Human serum anti-Neu5Gc antibodies could bind the Neu5Gc-containing Cetuximab and generate immune complexes, but did not bind Panitumumab. Furthermore, consistent with their expected immunogenic properties, injection of these drugs to the human-like Neu5Gc-deficient *Cmah*-KO mice induced serum anti-Neu5Gc antibody only in the Neu5Gc-containing Cetuximab-treated group. In these mice, circulating serum anti-Neu5Gc antibodies resulted in a rapid clearance of the Neu5Gc-containing Cetuximab, but not of Panitumumab (61). Together, these data suggest that Neu5Gc on biotherapeutics could potentially affect drug levels in circulation through immune complex formation (Figure 2), at least in human-like mice. Currently, there is no evidence of drug neutralizing activity of anti-Neu5Gc antibodies. It remains to be investigated whether Neu5Gc/anti-Neu5Gc could affect drug clearance in patients, hence alter drug efficacy and as such play a role in the variability of the clinical responses observed across a population for a given biotherapeutic.

## Conclusions and Perspective

Biotherapeutics have revolutionized the treatment for numerous clinical conditions, yet immunogenicity and efficacy issues remain to be addressed. Currently, most biotherapeutics are produced in non-human mammalian cells to allow human-like glycosylation, as it was shown to dramatically affect pharmacological properties of these products. Yet, despite the fact that it was recognized that humans cannot produce the non-human carbohydrate Neu5Gc, its immunogenic potential was much ignored, and accordingly its expression on biotherapeutics was largely overlooked. In fact, non-human mammals produce Neu5Gc-glycans, against which all humans have circulating polyclonal antibodies. Moreover, Neu5Gc can be metabolized by human cells and become presented on cell surface glycans, glycoproteins and glycolipids. In addition, all humans examined thus far had serum anti-Neu5Gc responses at variable levels and repertoires. Neu5Gc on biotherapeutics may induce the pre-existing anti-Neu5Gc responses in humans, and these could potentially contribute to increased xenosialitis and related diseases, yet further evidence is needed to fully understand the developed responses and their effects in humans. Drug-induced or pre-existing anti-Neu5Gc antibody responses could potentially contribute to drug clearance from circulation through immune complex formation, thereby reducing drug efficacy, although clear evidence in humans is yet to be provided. While not discussed in this review, similar effects could be expected by αGal glycosylation on biotherapeutics since all humans have circulating anti-Gal antibodies. Thus, much of the mechanistic insights into the outcome of the co-existence of anti-Neu5Gc antibodies and antigenic Neu5Gc-containing biotherapeutics (or anti-Gal antibodies and antigenic αGal-containing biotherapeutics) in humans is largely unknown and warrants further investigation.

## Author Contributions

SY and VP-K wrote the manuscript.

### Conflict of Interest

The authors declare that the research was conducted in the absence of any commercial or financial relationships that could be construed as a potential conflict of interest.

## References

[1] Lalonde ME Durocher Y. Therapeutic glycoprotein production in mammalian cells. J Biotechnol. (2017) 251:128–40. 10.1016/j.jbiotec.2017.04.028 28465209

[2] Ghaderi D Zhang M Hurtado-Ziola N Varki A. Production platforms for biotherapeutic glycoproteins. Occurrence, impact, and challenges of non-human sialylation. Biotechnol Genet Eng Rev. (2012) 28:147–75. 10.5661/bger-28-147 22616486

[3] Rini JM Esko JD Varki A Chapter 6: Glycosyl transferases and glycan-processing enzymes. In: Varki A Cummings RD Esko JD Freeze HH Stanley P Bertozzi CR Hart GW Etzler ME, editors. Essentials of Glycobiology. 2nd ed. Cold Spring Harbor, NY: Cold Spring Harbor Laboratory Press (2009).

[4] Henrissat B Surolia A Stanley P Chapter 8: A genomic view of glycobiology. In: Varki A Cummings RD Esko JD Stanley P Hart GW Aebi M Darvill AG Kinoshita T Packer NH Prestegard JH Schnaar RL Seeberger PH, editors. Essentials of Glycobiology. Cold Spring Harbor, NY: Cold Spring Harbor Laboratory Press (2015). 28876809

[5] Paul A Padler-Karavani V. Evolution of sialic acids: implications in xenotransplant biology. Xenotransplantation. (2018) 25:e12424. 10.1111/xen.12424 29932472 PMC6756921

[6] Cummings RD Pierce JM. The challenge and promise of glycomics. Chem Biol. (2014) 21:1–15. 10.1016/j.chembiol.2013.12.010 24439204 PMC3955176

[7] Arnold JN Wormald MR Sim RB Rudd PM Dwek RA. The impact of glycosylation on the biological function and structure of human immunoglobulins. Annu Rev Immunol. (2007) 25:21–50. 10.1146/annurev.immunol.25.022106.141702 17029568

[8] Durocher Y Butler M. Expression systems for therapeutic glycoprotein production. Curr Opin Biotechnol. (2009) 20:700–7. 10.1016/j.copbio.2009.10.008 19889531

[9] Higgins E. Carbohydrate analysis throughout the development of a protein therapeutic. Glycoconj J. (2009) 27:211–25. 10.1007/s10719-009-9261-x 19888650 PMC2821524

[10] Jennewein MF Alter G. The immunoregulatory roles of antibody glycosylation. Trends Immunol. (2017) 38:358–72. 10.1016/j.it.2017.02.004 28385520

[11] Liu L. Pharmacokinetics of monoclonal antibodies and Fc-fusion proteins. Protein Cell. (2018) 9:15–32. 10.1007/s13238-017-0408-4 28421387 PMC5777971

[12] Higel F Sandl T Kao CY Pechinger N Sörgel F Friess W. N-glycans of complex glycosylated biopharmaceuticals and their impact on protein clearance. Eur J Pharm Biopharm. (2019) 139:123–31. 10.1016/j.ejpb.2019.03.018 30905778

[13] Kontoravdi C del Val IJ Computational tools for predicting and controlling the glycosylation of biopharmaceuticals. Curr Opin Chem Eng. (2018) 22:89–97. 10.1016/j.coche.2018.08.007

[14] Bork K Horstkorte R Weidemann W. Increasing the sialylation of therapeutic glycoproteins: the potential of the sialic acid biosynthetic pathway. J Pharm Sci. (2009) 98:3499–508. 10.1002/jps.21684 19199295

[15] Saunders KO. Conceptual approaches to modulating antibody effector functions and circulation half-life. Front Immunol. (2019) 10:1296. 10.3389/fimmu.2019.01296 31231397 PMC6568213

[16] Weiss P Ashwell G. The asialoglycoprotein receptor: properties and modulation by ligand. Prog Clin Biol Res. (1989) 300:169–84. 2674962

[17] Tsuda E Kawanishi G Ueda M Masuda S Sasaki R. The role of carbohydrate in recombinant human erythropoietin. Eur J Biochem. (1990) 188:405–11. 10.1111/j.1432-1033.1990.tb15417.x 2156701

[18] Lawson EQ Hedlund BE Ericson ME Mood DA Litman GW Middaugh R. Effect of carbohydrate on protein solubility. Arch Biochem Biophys. (1983) 220:572–5. 10.1016/0003-9861(83)90449-6 6824341

[19] Vidarsson G Dekkers G Rispens T. IgG subclasses and allotypes: from structure to effector functions. Front Immunol. (2014) 5:520. 10.3389/fimmu.2014.00520 25368619 PMC4202688

[20] de Taeye SW Rispens T Vidarsson G. The ligands for human IgG and their effector functions. Antibodies. (2019) 8:30. 10.3390/antib8020030 31544836 PMC6640714

[21] Jefferis R. Glycosylation of recombinant antibody therapeutics. Biotechnol Prog. (2005) 21:11–6. 10.1021/bp040016j 15903235

[22] Qian J Liu T Yang L Daus A Crowley R Zhou Q. Structural characterization of N-linked oligosaccharides on monoclonal antibody cetuximab by the combination of orthogonal matrix-assisted laser desorption/ionization hybrid quadrupole-quadrupole time-of-flight tandem mass spectrometry and sequential enzymatic digestion. Anal Biochem. (2007) 364:8–18. 10.1016/j.ab.2007.01.023 17362871

[23] Dahal LN Roghanian A Beers SA Cragg MS. FcγR requirements leading to successful immunotherapy. Immunol Rev. (2015) 268:104–22. 10.1111/imr.12342 26497516

[24] Oliva KD Cavanaugh JM Cobb BA. Antibody receptors steal the sweet spotlight. J Biol Chem. (2018) 293:3490–1. 10.1074/jbc.H118.001955 29523693 PMC5846142

[25] Goulabchand R Vincent T Batteux F Eliaou JF Guilpain P. Impact of autoantibody glycosylation in autoimmune diseases. Autoimmun Rev. (2014) 13:742–50. 10.1016/j.autrev.2014.02.005 24657512

[26] Zhang D Chen B Wang Y Xia P He C Liu Y. Disease-specific IgG Fc N-glycosylation as personalized biomarkers to differentiate gastric cancer from benign gastric diseases. Sci Rep. (2016) 6:25957. 10.1038/srep25957 27173519 PMC4865947

[27] Tanaka T Yoneyama T Noro D Imanishi K Kojima Y Hatakeyama S. Aberrant N-glycosylation profile of serum immunoglobulins is a diagnostic biomarker of urothelial carcinomas. Int J Mol Sci. (2017) 18:2632. 10.3390/ijms18122632 29210993 PMC5751235

[28] Theodoratou E Thaçi K Agakov F Timofeeva MN Štambuk J Pučić-Baković M. Glycosylation of plasma IgG in colorectal cancer prognosis. Sci Rep. (2016) 6:28098. 10.1038/srep28098 27302279 PMC4908421

[29] Alter G Ottenhoff THM Joosten SA. Antibody glycosylation in inflammation, disease and vaccination. Semin Immunol. (2018) 39:102–10. 10.1016/j.smim.2018.05.003 29903548 PMC8731230

[30] Jefferis R. Glycosylation as a strategy to improve antibody-based therapeutics. Nat Rev Drug Discov. (2009) 8:226–34. 10.1038/nrd2804 19247305

[31] Shinkawa T Nakamura K Yamane N Shoji-Hosaka E Kanda Y Sakurada M. The absence of fucose but not the presence of galactose or bisecting N-acetylglucosamine of human IgG1 complex-type oligosaccharides shows the critical role of enhancing antibody-dependent cellular cytotoxicity. J Biol Chem. (2003) 278:3466–73. 10.1074/jbc.M210665200 12427744

[32] Raju TS. Terminal sugars of Fc glycans influence antibody effector functions of IgGs. Curr Opin Immunol. (2008) 20:471–8. 10.1016/j.coi.2008.06.007 18606225

[33] Malhotra R Wormald MR Rudd PM Fischer PB Dwek RA Sim RB. Glycosylation changes of IgG associated with rheumatoid arthritis can activate complement via the mannose-binding protein. Nat Med. (1995) 1:237–43. 10.1038/nm0395-237 7585040

[34] Kaneko Y Nimmerjahn F Ravetch JV. Anti-inflammatory activity of immunoglobulin G resulting from Fc sialylation. Science. (2006) 313:670–3. 10.1126/science.1129594 16888140

[35] Buettner MJ Shah SR Saeui CT Ariss R Yarema KJ. Improving immunotherapy through glycodesign. Front Immunol. (2018) 9:2485. 10.3389/fimmu.2018.02485 30450094 PMC6224361

[36] Morgan H Tseng SY Gallais Y Leineweber M Buchmann P Riccardi S. Evaluation of *in vitro* assays to assess the modulation of dendritic cells functions by therapeutic antibodies and aggregates. Front Immunol. (2019) 10:601. 10.3389/fimmu.2019.00601 31001248 PMC6455063

[37] Dumont J Euwart D Mei B Estes S Kshirsagar R. Human cell lines for biopharmaceutical manufacturing: history, status, and future perspectives. Crit Rev Biotechnol. (2016) 36:1110–22. 10.3109/07388551.2015.1084266 26383226 PMC5152558

[38] Tejwani V Andersen MR Nam JH Sharfstein ST. Glycoengineering in CHO cells: advances in systems biology. Biotechnol J. (2018) 13:e1700234. 10.1002/biot.201700234 29316325

[39] Blondeel EJM Aucoin MG. Supplementing glycosylation: a review of applying nucleotide-sugar precursors to growth medium to affect therapeutic recombinant protein glycoform distributions. Biotechnol Adv. (2018) 36:1505–23. 10.1016/j.biotechadv.2018.06.008 29913209

[40] Fischer S Handrick R Otte K. The art of CHO cell engineering: a comprehensive retrospect and future perspectives. Biotechnol Adv. (2015) 33:1878–96. 10.1016/j.biotechadv.2015.10.015 26523782

[41] Könitzer JD Müller MM Leparc G Pauers M Bechmann J Schulz P. A global RNA-seq-driven analysis of CHO host and production cell lines reveals distinct differential expression patterns of genes contributing to recombinant antibody glycosylation. Biotechnol J. (2015) 10:1412–23. 10.1002/biot.201400652 26212696

[42] Richelle A Lewis NE. Improvements in protein production in mammalian cells from targeted metabolic engineering. Curr Opin Syst Biol. (2017) 6:1–6. 10.1016/j.coisb.2017.05.019 29104947 PMC5663301

[43] Chiang AW Li S Spahn PN Richelle A Kuo CC Samoudi M. Modulating carbohydrate-protein interactions through glycoengineering of monoclonal antibodies to impact cancer physiology. Curr Opin Struct Biol. (2016) 40:104–11. 10.1016/j.sbi.2016.08.008 27639240 PMC5161599

[44] Spahn PN Lewis NE. Systems glycobiology for glycoengineering. Curr Opin Biotechnol. (2014) 30:218–24. 10.1016/j.copbio.2014.08.004 25202878

[45] Padler-Karavani V Varki A. Potential impact of the non-human sialic acid N-glycolylneuraminic acid on transplant rejection risk. Xenotransplantation. (2011) 18:1–5. 10.1111/j.1399-3089.2011.00622.x 21342282 PMC3098739

[46] Galili U. Natural anti-carbohydrate antibodies contributing to evolutionary survival of primates in viral epidemics. Glycobiology. (2016) 26:1140–50. 10.1093/glycob/cww088 27567275

[47] Padler-Karavani V Yu H Cao H Chokhawala H Karp F Varki N. Diversity in specificity, abundance, and composition of anti-Neu5Gc antibodies in normal humans: potential implications for disease. Glycobiology. (2008) 18:818–30. 10.1093/glycob/cwn072 18669916 PMC2586336

[48] Altman MO Gagneux P. Absence of Neu5Gc and presence of Anti-Neu5Gc antibodies in humans-an evolutionary perspective. Front Immunol. (2019) 10:789. 10.3389/fimmu.2019.00789 31134048 PMC6524697

[49] Leviatan Ben-Arye S Yu H Chen X Padler-Karavani V Profiling Anti-Neu5Gc IgG in human sera with a sialoglycan microarray assay. J Vis Exp. (2017) 125:56094 10.3791/56094 PMC5612302 28745644

[50] Tangvoranuntakul P Gagneux P Diaz S Bardor M Varki N Varki A. Human uptake and incorporation of an immunogenic nonhuman dietary sialic acid. Proc Natl Acad Sci USA. (2003) 100:12045–50. 10.1073/pnas.2131556100 14523234 PMC218710

[51] Nguyen DH Tangvoranuntakul P Varki A. Effects of natural human antibodies against a nonhuman sialic acid that metabolically incorporates into activated and malignant immune cells. J Immunol. (2005) 175:228–36. 10.4049/jimmunol.175.1.228 15972653

[52] Samraj AN Läubli H Varki N Varki A Involvement of a non-human sialic acid in human cancer. Front Oncol. (2014) 4:33 10.3389/fonc.2014.00033 24600589 PMC3928833

[53] Dhar C Sasmal A Varki A. From “serum sickness” to “xenosialitis”: past, present, and future significance of the non-human sialic acid Neu5Gc. Front Immunol. (2019) 10:807. 10.3389/fimmu.2019.00807 31057542 PMC6481270

[54] Varki A. Are humans prone to autoimmunity? Implications from evolutionary changes in hominin sialic acid biology. J Autoimmun. (2017) 83:134–42. 10.1016/j.jaut.2017.07.011 28755952

[55] Alisson-Silva F Kawanishi K Varki A. Human risk of diseases associated with red meat intake: analysis of current theories and proposed role for metabolic incorporation of a non-human sialic acid. Mol Aspects Med. (2016) 51:16–30. 10.1016/j.mam.2016.07.002 27421909 PMC5035214

[56] Samraj AN Pearce OM Läubli H Crittenden AN Bergfeld AK Banda K. A red meat-derived glycan promotes inflammation and cancer progression. Proc Natl Acad Sci USA. (2015) 112:542–7. 10.1073/pnas.1417508112 25548184 PMC4299224

[57] Okerblom J Varki A. Biochemical, cellular, physiological, and pathological consequences of human loss of N-glycolylneuraminic acid. Chembiochem. (2017) 18:1155–71. 10.1002/cbic.201700077 28423240

[58] Kooner AS Yu H Chen X. Synthesis of N-glycolylneuraminic acid (Neu5Gc) and its glycosides. Front Immunol. (2019) 10:2004. 10.3389/fimmu.2019.02004 31555264 PMC6724515

[59] McQuillan AM Byrd-Leotis L Heimburg-Molinaro J Cummings RD. Natural and synthetic sialylated glycan microarrays and their applications. Front Mol Biosci. (2019) 6:88. 10.3389/fmolb.2019.00088 31572731 PMC6753469

[60] Breimer ME Holgersson J. The structural complexity and animal tissue distribution of N-glycolylneuraminic acid (Neu5Gc)-terminated glycans. Implications for their immunogenicity in clinical xenografting. Front Mol Biosci. (2019) 6:57. 10.3389/fmolb.2019.00057 31428616 PMC6690001

[61] Ghaderi D Taylor RE Padler-Karavani V Diaz S Varki A. Implications of the presence of N-glycolylneuraminic acid in recombinant therapeutic glycoproteins. Nat Biotechnol. (2010) 28:863–7. 10.1038/nbt.1651 20657583 PMC3077421

[62] Couvrat-Desvergnes G Salama A Le Berre L Evanno G Viklicky O Hruba P. Rabbit antithymocyte globulin-induced serum sickness disease and human kidney graft survival. J Clin Invest. (2015) 125:4655–65. 10.1172/JCI82267 26551683 PMC4665787

[63] Amon R Ben-Arye SL Engler L Yu H Lim N Berre LL. Glycan microarray reveal induced IgGs repertoire shift against a dietary carbohydrate in response to rabbit anti-human thymocyte therapy. Oncotarget. (2017) 8:112236–44. 10.18632/oncotarget.23096 29348821 PMC5762506

[64] Mai HL Treilhaud M Ben-Arye SL Yu H Perreault H Ang E. Poor patient and graft outcome after induction treatment by antithymocyte globulin in recipients of a kidney graft after nonrenal organ transplantation. Transplant Direct. (2018) 4:e357. 10.1097/TXD.0000000000000772 29707628 PMC5908458

[65] Le Berre L Danger R Mai HL Amon R Leviatan Ben-Arye S Bruneau S. Elicited and pre-existing anti-Neu5Gc antibodies differentially affect human endothelial cells transcriptome. Xenotransplantation. (2019) 26:e12535. 10.1111/xen.12535 31293002

[66] Angata T Varki A. Chemical diversity in the sialic acids and related alpha-keto acids: an evolutionary perspective. Chem Rev. (2002) 102:439–69. 10.1021/cr000407m 11841250

[67] Varki A. Loss of N-glycolylneuraminic acid in humans: mechanisms, consequences, and implications for hominid evolution. Am J Phys Anthropol Suppl. (2001) 33:54–69. 10.1002/ajpa.10018 11786991 PMC7159735

[68] Chou HH Takematsu H Diaz S Iber J Nickerson E Wright KL. A mutation in human CMP-sialic acid hydroxylase occurred after the Homo-Pan divergence. Proc Natl Acad Sci USA. (1998) 95:11751–6. 10.1073/pnas.95.20.11751 9751737 PMC21712

[69] Hayakawa T Satta Y Gagneux P Varki A Takahata N. Alu-mediated inactivation of the human CMP- N-acetylneuraminic acid hydroxylase gene. Proc Natl Acad Sci USA. (2001) 98:11399–404. 10.1073/pnas.191268198 11562455 PMC58741

[70] Chou HH Hayakawa T Diaz S Krings M Indriati E Leakey M. Inactivation of CMP-N-acetylneuraminic acid hydroxylase occurred prior to brain expansion during human evolution. Proc Natl Acad Sci USA. (2002) 99:11736–41. 10.1073/pnas.182257399 12192086 PMC129338

[71] Malykh YN Schauer R Shaw L. N-Glycolylneuraminic acid in human tumours. Biochimie. (2001) 83:623–34. 10.1016/S0300-9084(01)01303-7 11522391

[72] Bardor M Nguyen DH Diaz S Varki A. Mechanism of uptake and incorporation of the non-human sialic acid N-glycolylneuraminic acid into human cells. J Biol Chem. (2005) 280:4228–37. 10.1074/jbc.M412040200 15557321

[73] Banda K Gregg CJ Chow R Varki NM Varki A. Metabolism of vertebrate amino sugars with N-glycolyl groups: mechanisms underlying gastrointestinal incorporation of the non-human sialic acid xeno-autoantigen n-glycolylneuraminic acid. J Biol Chem. (2012) 287:28852–64. 10.1074/jbc.M112.364182 22692204 PMC3436511

[74] Bergfeld AK Pearce OM Diaz SL Pham T Varki A. Metabolism of vertebrate amino sugars with N-glycolyl groups: elucidating the intracellular fate of the non-human sialic acid n-glycolylneuraminic acid. J Biol Chem. (2012) 287:28865–81. 10.1074/jbc.M112.363549 22692205 PMC3436522

[75] Amon R Reuven EM Leviatan Ben-Arye S Padler-Karavani V. Glycans in immune recognition and response. Carbohydr Res. (2014) 389:115–22. 10.1016/j.carres.2014.02.004 24680512

[76] Zhu A Hurst R. Anti-N-glycolylneuraminic acid antibodies identified in healthy human serum. Xenotransplantation. (2002) 9:376–81. 10.1034/j.1399-3089.2002.02138.x 12371933

[77] Padler-Karavani V Hurtado-Ziola N Pu M Yu H Huang S Muthana S. Human xeno-autoantibodies against a non-human sialic acid serve as novel serum biomarkers and immunotherapeutics in cancer. Cancer Res. (2011) 71:3352–63. 10.1158/0008-5472.CAN-10-4102 21505105 PMC3085609

[78] Leviatan Ben-Arye S Schneider C Yu H Bashir S Chen X von Gunten S Differential recognition of diet-derived Neu5Gc-neoantigens on glycan microarrays by carbohydrate-specific pooled human IgG and IgA antibodies. Bioconjug Chem. (2019) 30:1565–74. 10.1021/acs.bioconjchem.9b00273 30994337 PMC6756923

[79] Lu Q Padler-Karavani V Yu H Chen X Wu SL Varki A. LC-MS analysis of polyclonal human anti-Neu5Gc xeno-autoantibodies immunoglobulin G Subclass and partial sequence using multistep intravenous immunoglobulin affinity purification and multienzymatic digestion. Anal Chem. (2012) 84:2761–8. 10.1021/ac2030893 22390546 PMC4142218

[80] Taylor RE Gregg CJ Padler-Karavani V Ghaderi D Yu H Huang S. Novel mechanism for the generation of human xeno-autoantibodies against the nonhuman sialic acid N-glycolylneuraminic acid. J Exp Med. (2010) 207:1637–46. 10.1084/jem.20100575 20624889 PMC2916132

[81] Salama A Mosser M Lévêque X Perota A Judor JP Danna C Neu5Gc and α1-3 GAL xenoantigen knockout does not affect glycemia homeostasis and insulin secretion in pigs. Diabetes. (2017) 66:987–93. 10.2337/db16-1060 28082457

[82] Padler-Karavani V Tremoulet AH Yu H Chen X Burns JC Varki A. A simple method for assessment of human anti-Neu5Gc antibodies applied to Kawasaki disease. PLoS ONE. (2013) 8:e58443. 10.1371/journal.pone.0058443 23520510 PMC3592828

[83] Scobie L Padler-Karavani V Le Bas-Bernardet S Crossan C Blaha J Matouskova M. Long-term IgG response to porcine Neu5Gc antigens without transmission of PERV in burn patients treated with porcine skin xenografts. J Immunol. (2013) 191:2907–15. 10.4049/jimmunol.1301195 23945141 PMC3782708

[84] Samraj AN Bertrand KA Luben R Khedri Z Yu H Nguyen D. Polyclonal human antibodies against glycans bearing red meat-derived non-human sialic acid N-glycolylneuraminic acid are stable, reproducible, complex and vary between individuals: Total antibody levels are associated with colorectal cancer risk. PLoS ONE. (2018) 13:e0197464. 10.1371/journal.pone.0197464 29912879 PMC6005533

[85] Hedlund M Padler-Karavani V Varki NM Varki A. Evidence for a human-specific mechanism for diet and antibody-mediated inflammation in carcinoma progression. Proc Natl Acad Sci USA. (2008) 105:18936–41. 10.1073/pnas.0803943105 19017806 PMC2596253

[86] Pham T Gregg CJ Karp F Chow R Padler-Karavani V Cao H. Evidence for a novel human-specific xeno-auto-antibody response against vascular endothelium. Blood. (2009) 114:5225–35. 10.1182/blood-2009-05-220400 19828701 PMC2792214

[87] Ma Y Yang W Li T Liu Y Simon TG Sui J. Meat intake and risk of hepatocellular carcinoma in two large US prospective cohorts of women and men. Int J Epidemiol. (2019) 48:1863–71. 10.1093/ije/dyz146 31302687 PMC7967811

[88] Hammerling U Bergman Laurila J Grafström R Ilbäck NG. Consumption of red/processed meat and colorectal carcinoma: possible mechanisms underlying the significant association. Crit Rev Food Sci Nutr. (2016) 56:614–34. 10.1080/10408398.2014.972498 25849747

[89] Cho E Chen WY Hunter DJ Stampfer MJ Colditz GA Hankinson SE. Red meat intake and risk of breast cancer among premenopausal women. Arch Intern Med. (2006) 166:2253–9. 10.1001/archinte.166.20.2253 17101944

[90] Kawanishi K Dhar C Do R Varki N Gordts PLSM Varki A. Human species-specific loss of CMP-N-acetylneuraminic acid hydroxylase enhances atherosclerosis via intrinsic and extrinsic mechanisms. Proc Natl Acad Sci USA. (2019) 116:16036–45. 10.1073/pnas.1902902116 31332008 PMC6690033

[91] Micha R Wallace SK Mozaffarian D. Red and processed meat consumption and risk of incident coronary heart disease, stroke, and diabetes mellitus: a systematic review and meta-analysis. Circulation. (2010) 121:2271–83. 10.1161/CIRCULATIONAHA.109.924977 20479151 PMC2885952

[92] Tong TYN Appleby PN Bradbury KE Perez-Cornago A Travis RC Clarke R. Risks of ischaemic heart disease and stroke in meat eaters, fish eaters, and vegetarians over 18 years of follow-up: results from the prospective EPIC-Oxford study. BMJ. (2019) 366:l4897. 10.1136/bmj.l4897 31484644 PMC6724406

[93] Soulillou JP. Missing links in multiple sclerosis etiology. A working connecting hypothesis. Med Hypotheses. (2013) 80:509–16. 10.1016/j.mehy.2013.01.036 23466062

[94] Varki NM Strobert E Dick EJ Benirschke K Varki A. Biomedical differences between human and nonhuman hominids: potential roles for uniquely human aspects of sialic acid biology. Annu Rev Pathol. (2011) 6:365–93. 10.1146/annurev-pathol-011110-130315 21073341

[95] Yuen CT Storring PL Tiplady RJ Izquierdo M Wait R Gee CK. Relationships between the N-glycan structures and biological activities of recombinant human erythropoietins produced using different culture conditions and purification procedures. Br J Haematol. (2003) 121:511–26. 10.1046/j.1365-2141.2003.04307.x 12716378

[96] Noguchi A Mukuria CJ Suzuki E Naiki M. Immunogenicity of N-glycolylneuraminic acid-containing carbohydrate chains of recombinant human erythropoietin expressed in Chinese hamster ovary cells. J Biochem. (1995) 117:59–62. 10.1093/oxfordjournals.jbchem.a124721 7539788

[97] Yoo EM Chintalacharuvu KR Penichet ML Morrison SL. Myeloma expression systems. J Immunol Methods. (2002) 261:1–20. 10.1016/S0022-1759(01)00559-2 11861062

[98] Muchmore EA Milewski M Varki A Diaz S. Biosynthesis of N-glycolyneuraminic acid. The primary site of hydroxylation of N-acetylneuraminic acid is the cytosolic sugar nucleotide pool. J Biol Chem. (1989) 264:20216–23. 2684973

[99] Reuven EM Leviatan Ben-Arye S Marshanski T Breimer ME Yu H Fellah-Hebia I. Characterization of immunogenic Neu5Gc in bioprosthetic heart valves. Xenotransplantation. (2016) 23:381–92. 10.1111/xen.12260 27610947 PMC5036590

[100] Burdorf L Azimzadeh AM Pierson RN. Progress and challenges in lung xenotransplantation: an update. Curr Opin Organ Transplant. (2018) 23:621–7. 10.1097/MOT.0000000000000582 30234737

[101] French BM Sendil S Pierson RN Azimzadeh AM. The role of sialic acids in the immune recognition of xenografts. Xenotransplantation. (2017) 24:12345. 10.1111/xen.12345 29057592 PMC10167934

[102] Byrne GW McGregor CGA Breimer ME. Recent investigations into pig antigen and anti-pig antibody expression. Int J Surg. (2015) 23:223–8. 10.1016/j.ijsu.2015.07.724 26306769 PMC4684721

[103] Perota A Lagutina I Duchi R Zanfrini E Lazzari G Judor JP. Generation of cattle knockout for galactose-α1,3-galactose and N-glycolylneuraminic acid antigens. Xenotransplantation. (2019) 26:e12524. 10.1111/xen.12524 31115108 PMC6852128

[104] Fischer K Kraner-Scheiber S Petersen B Rieblinger B Buermann A Flisikowska T. Efficient production of multi-modified pigs for xenotransplantation by ‘combineering', gene stacking and gene editing. Sci Rep. (2016) 6:29081. 10.1038/srep29081 27353424 PMC4926246

[105] Zhang R Wang Y Chen L Wang R Li C Li X. Reducing immunoreactivity of porcine bioprosthetic heart valves by genetically-deleting three major glycan antigens, GGTA1/β4GalNT2/CMAH. Acta Biomater. (2018) 72:196–205. 10.1016/j.actbio.2018.03.055 29631050

[106] Kemter E Denner J Wolf E. Will genetic engineering carry xenotransplantation of pig islets to the clinic. Curr Diab Rep. (2018) 18:103. 10.1007/s11892-018-1074-5 30229378

[107] Cimeno A Hassanein W French BM Powell JM Burdorf L Goloubeva O. N-glycolylneuraminic acid knockout reduces erythrocyte sequestration and thromboxane elaboration in an *ex vivo* pig-to-human xenoperfusion model. Xenotransplantation. (2017) 24:12339. 10.1111/xen.12339 28940313 PMC6687465

[108] Salama A Evanno G Lim N Rousse J Le Berre L Nicot A Anti-Gal and anti-Neu5Gc responses in nonimmunosuppressed patients following treatment with rabbit anti-thymocyte polyclonal IgGs. Transplantation. (2017) 101:2501–7. 10.1097/TP.0000000000001686 28198767 PMC12407183

[109] Yu C Gao K Zhu L Wang W Wang L Zhang F At least two Fc Neu5Gc residues of monoclonal antibodies are required for binding to anti-Neu5Gc antibody. Sci Rep. (2016) 7:20029 10.1038/srep20029 26823113 PMC4731815

[110] Rousse J Salama A Leviatan Ben-Arye S Hruba P Slatinska J Evanno G. Quantitative and qualitative changes in anti-Neu5Gc antibody response following rabbit anti-thymocyte IgG induction in kidney allograft recipients. Eur J Clin Invest. (2019) 49:e13069. 10.1111/eci.13069 30620396

[111] Soulillou JP Süsal C Döhler B Opelz G. No increase in colon cancer risk following induction with Neu5Gc-bearing rabbit anti-T cell IgG (ATG) in recipients of kidney transplants. Cancers. (2018) 10:E324. 10.3390/cancers10090324 30213027 PMC6162487

[112] Noguchi A Mukuria CJ Suzuki E Naiki M. Failure of human immunoresponse to N-glycolylneuraminic acid epitope contained in recombinant human erythropoietin. Nephron. (1996) 72:599–603. 10.1159/000188946 8730428

[113] Mohty M. Mechanisms of action of antithymocyte globulin: T-cell depletion and beyond. Leukemia. (2007) 21:1387–94. 10.1038/sj.leu.2404683 17410187

[114] Gitelman SE Gottlieb PA Rigby MR Felner EI Willi SM Fisher LK. Antithymocyte globulin treatment for patients with recent-onset type 1 diabetes: 12-month results of a randomised, placebo-controlled, phase 2 trial. Lancet Diabetes Endocrinol. (2013) 1:306–16. 10.1016/S2213-8587(13)70065-2 24622416 PMC6489466

[115] Bashir S Leviatan Ben Arye S Reuven EM Yu H Costa C Galiñanes M. Presentation mode of glycans affect recognition of human serum anti-Neu5Gc IgG antibodies. Bioconjug Chem. (2019) 30:161–8. 10.1021/acs.bioconjchem.8b00817 30500162 PMC6768799

